# Characteristics and Results of the Management of Diffuse Large B-Cell Lymphomas: The Experience of Côte d'Ivoire

**DOI:** 10.1155/2012/945138

**Published:** 2012-05-28

**Authors:** Aïssata Tolo Diebkilé, Boidy Kouakou, Emeraude N'dhatz, Clotaire D. Nanho, N'Dogomo Meité, Roméo Ayémou, Mamadou Y. Sekongo, Paul Kouéhion, Mozart Konan, Gustave K. Koffi, Ibrahima Sanogo

**Affiliations:** Department of Clinical Hematology, Yopougon Teaching Hospital, P.O. Box 632, Abidjan 21, Cote d'Ivoire

## Abstract

Diffuse large B-cell lymphomas have been little studied in black Africans. The purpose of our study was to determine the characteristics and results of the management of these lymphomas. *Patients and Methods*. In a descriptive and analytic retrospective study we studied the medical records of 63 patients with diffuse large B-cell lymphoma hospitalized during the period from 1991 to 2007. The diagnosis was made after lymph node or organ biopsy. Response to treatment, OS, PFS, and toxicity were studied. The complete response has been analyzed univariate and multivariate analysis. *Results*. The median age was 42 years. The sex ratio was 2. The HIV serology was positive in 11 cases, and 8 patients had antiretroviral therapy. In 71% the lymphoma was at stages III and IV of Ann Arbor. IPI was ≥3 in 65%. Complete remission was achieved in 43%. Only 43% of patients had had a good compliance. Progression-free survival at 3 years was 32%, and overall survival at 3 years was 50%. 13% of patients were lost to follow up, and 51% of them died. In terms of analysis the complete remission rate was influenced by the stage of Ann Arbor (*P* < 0.0001), biological b symptoms (*P* < 0.01), the IPI (*P* < 0.0001), and the socioeconomic standing (*P* = 0.001). In multivariate analysis, only IPI and stage of Ann Arbor influence the complete remission.

## 1. Introduction

Diffuse large B-cell lymphomas are clonal proliferations of B-lymphocytes. It is the most common form of non-Hodgkin's lymphomas accounting for 30 to 40% of lymphomas in adults [1, 2]. They are aggressive because the cells are young and express the proliferation marker Ki 67+. Thus, the doubling of the tumor mass is rapid, in the order of 15 days to one month. Diffuse large B-cell lymphomas constitute a heterogenous group with at least 15 entities according to the 2008 World Health Organization (WHO) classification and two molecular subtypes: those of germinal center type and those of activated B-cells type [3, 4]. These lymphomas may originate in the lymph nodes or in extranodal sites; they can be localized or disseminated.

In terms of therapy, these lymphomas are usually sensitive to chemotherapy including CHOP protocol (Cyclophosphamide, Doxorubicin, Vincristine, and Prednisone), which has been used since 1973 [5]. Then appeared the 3rd and 2nd generation protocols, which improved the complete remission rate. At the moment, Rituximab combined with CHOP, used in the first line, has further improved complete remission rate and survival. This treatment is adapted to the Internal Prognostic Index (IPI), which consists of 5 factors: age, performance score, stage of Ann Arbor, LDH rate, and extra-nodal affection. These diffuse large B-cell lymphomas have been little studied in black Africans. The purpose of our study was to determine the results of the management of these lymphomas.

## 2. Patients and Methods

Our study was carried out in the Department of Clinical Hematology of Yopougon Teaching Hospital of Abidjan, Côte d'Ivoire from July 1991 to September 2007. Patients hospitalized with diffuse large B-cell lymphoma of histological and immunohistochemical diagnosis (CD 19, CD 20, CD 22, and CD 79a) after lymph node or organ biopsy, with a complete medical record and who received CHOP protocol for treatment. Diffuse large B-cell lymphomas after transformation of a follicular lymphoma and primitive diffuse large B-cell lymphomas of the central nervous system were excluded. Sixty-three records were selected. The study was retrospective, descriptive, and analytic. Each medical record was operated using a survey form. The collection of data in the record looked for epidemiological parameters: age, sex, socioeconomic standing, and history of exposure to known etiologic factors. The clinical status took into account the general condition (weight loss, profuse night sudation, fever, and index of the activity of the WHO) as well as the various devices in the search for tumor lesion or other. Para-clinically, a lymph node or organ biopsy with pathological examination was performed. In each patient a cervicothoracic and abdominopelvic computed tomography (CT scan), a bone marrow biopsy, a lumbar puncture with cytological study of the cerebrospinal fluid, a check-up of biological course (blood count, fibrinemia, LDH, *β*2 microglobulin, protein electrophoresis, serum electrolytes, uric acid) and a pretherapeutic check-up (liver and kidney check-up, echocardiography, HIV status, and B and C viral serology) were performed. In terms of therapy, chemotherapy with CHOP protocol was assessed (6 to 9 cycles were planned) and prognostic factors were identified by univariate and multivariate analysis of the therapeutic response.

The results of midterm assessment of chemotherapy and postchemotherapy were clinical, biological, and radiological (CT scan).

Complete remission was characterized by the disappearance of all tumor sites confirmed by the results of assessment. Partial remission corresponded to a partial regression of the tumor and failure to an absence of remission.

Therapeutic compliance is defined by the regularity of chemotherapy administration. Patients who received treatment every 21 days had a good compliance; on the other hand, those who had the treatments with intervals superior than 21 days had a poor compliance.

The assessment of the socioeconomic level was done according to indirect criteria, which were occupation, number of children, and type of housing.

Overall survival (OS) was calculated from the date of biopsy to the time of death or to the time of the last follow-up. Progression-free survival (PFS) was calculated from the date of biopsy to the time of relapse, progression, death, or the last follow-up.

Our study has been approved by an Ethics Committee, and has been conducted in accordance with the Declaration of Helsinki. Appropriate written consent for the CHOP treatement was obtained.

The collected data were analyzed with the software program Epi-Info 6.04b and Stat view 5.0. The statistic analysis used the calculation of averages and the Khi2 test. The level of significance was set at 5% for the value of *P*. The calculation of survival was done according to Kaplan-Meir's method thanks to the existence in the record of a date of inclusion (date of admission) and a date of assessment (date of death or late news) mentioned by day, month, and year.

CHOP protocol is shown in Table 1.

## 3. Results

### 3.1. Descriptive Results

General characteristics (epidemiological, and clinical) are presented in Table 2, and biological characteristics of patients in Table 3.

Out of a total of 63 patients, the median age was 42 years with extremes of 12 and 75 years. The sex ratio was 2.

In 68% patients were referred to the Department of Clinical Hematology with their diagnosis of lymphoma. In 32% it was for polyadenopathy, or splenomegaly or hepatomegaly. The delay of consultation was less than 3 months in 24%, 3 to 6 months in 41%, and more than 6 months in 35%.

Concerning HIV infection, 11 patients (17%) were positive; 8 patients (13%) were on antiretroviral treatment with a rate of CD4 > 200/mm^3^. In 3 patients (6%) the rate of CD4 was <50/mm^3^.

As for the treatment (Table 4) the remission rate was 83%, with 43% of complete remission and 40% of partial remission. The rate of failure was 17%.

Toxicity related to the treatment was hematological (23 cases, i.e. 37%) and required hematological resuscitation and the administration of growth factor (G-CSF). Metabolic toxicity came into the second position with 20 cases, that is, 32%, with hyperkalemia and/or hyperphosphatemia and/or hypocalcemia and/or increased level of LDH. Other toxicities were brought out: digestive toxicity (7 cases i.e., 11%), cardiac toxicity (3 cases, i.e., 5%), neuropathies (3 cases, i.e., 5%), alopecia (2 cases, i.e., 3%).

The median follow-up duration was 3 years. Median OS was 49% and median PFS was 32%. They were, respectively, (OS and PFS) 78% and 74% at 3 years for patients with IPI at 0 and 1 and 29% and 26% for those with IPI ≥ 3.

Evolution to death was observed in 32 cases, that is, 51%. It was a progression of the disease in 17 cases (27%), and of hematological toxicity in 7 cases (11%), of infections in 4 cases (6%), of metabolic toxicity in 2 cases (3%), and in 2 cases (3%) the cause of death was undetermined. Seven patients out of 11 with HIV died.

**Figure 1:**
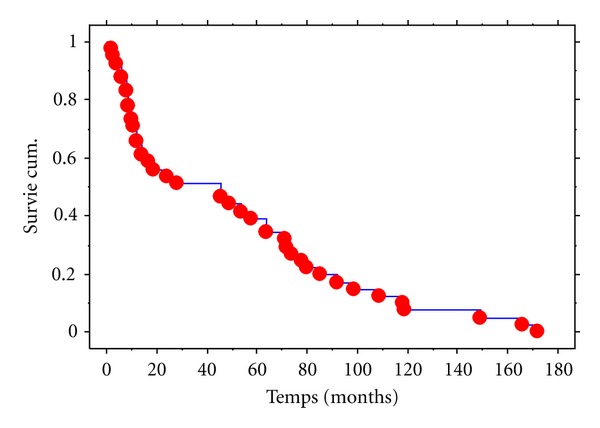
Overall survival curve of the patients with DLBCL. Kaplan-Meier Graphe de Survie Cum. Pour Colonne 1 Variable censure: <Sans>.

The overall survival study is represented in Figure 1, with the months on axis.

### 3.2. Analytic Results

The relationship between the therapeutic response and the general characteristics of patients is given in Table 5, with univariate analysis.

In multivariate analysis, only IPI (*P* = 0.001) and stage of Ann Arbor (*P* = 0.01) are significantly related to complete remission.

## 4. Discussion

In our study, patient's median age was 42 years with extremes of 12 and 75 years. Diffuse large B-cell lymphoma can occur at any age but most patients were less than 60 years. The median age of discovery is, however, variable depending on subtypes, on an average of 58 years for the forms of the germinal center, 66 years for the activated forms, and 35 years for mediastinal forms [4].

The low median age of our study could be an epidemiological factor particularity of diffuse large B-cell lymphoma in black Africans. Indeed, recent data in the United States suggest that the age of onset of diffuse large B-cell lymphomas was lower in black Americans [4] compared to Caucasians. The sex ratio was 2. This male predominance is confirmed by several authors in the west [6–8]. In 68% of cases patients were referred to the Department of Clinical Hematology with their diagnosis of lymphoma. This demonstrates the readiness of general practitioners to perform lymph node or organ biopsies when faced with any lymphadenopathy or suspicious lesion, but consultation period was most often long, between 3 and 6 months in 41% and more than 6 months in 35%. Long consultation periods are partly due to ignorance of patients who consult traditional doctors first before going to hospital. This delay in diagnosis is one of the arguments in favor of the spread of the disease. This is the reason why 71% of patients were at stages III and IV of Ann Arbor. But this rapid spread of the disease may be a clinical particularity of diffuse large B-cell lymphomas in the black Africans. Actually according to Flowers [4], black Americans present a more advanced stage of the disease (III and IV). This has been demonstrated in retrospective cohort studies [9, 10]. This particular clinical presentation is not related to HIV infection according to these authors.

In our study we could not draw any conclusion regarding HIV infection because serology was not performed in 64% of our patients. Nevertheless, it was positive in 11 patients out of 23, that is, 48%. There is ample evidence that HIV infection and immunosuppression resulting from it increases the risk of developing cancer in general and in particular lymphomas [11–13]. According to some authors [14, 15] lymphomas occurring during HIV infection are usually B-cell lymphomas that are particularly centroblastic, diffuse, histiocytic, or immunoblastic. Future studies will determine the impact of racial, geographical, and environmental factors on the clinical presentation of these diffuse large B-cell lymphomas. 75% of patients presented at least a sign of clinical B symptoms in our series whereas in western series, 1/3 of patients presented B symptoms. This is probably due to the delay in consultation and also to the disseminated character of the disease [4, 16–18].

In terms of therapy, diffuse large B-cell lymphomas are very chemosensitive and curative even in the disseminated forms [5]. Our patients had had CHOP 21 protocol, which has for a long time been the standard treatment of diffuse large B-cell lymphomas [4]. With this protocol, the global response was 83%. We obtained 27 cases of complete remission, that is, 43%, 25 cases of partial remission, that is, 40% and 11 cases of failure, that is, 17%. Our complete remission rate is below that of western, American, and Asian authors who have complete remission rates ranging from 60 to 80% on CHOP [2, 4, 5, 19]. This low rate of complete remission could be explained by the number of cycles received by patients on the one hand and on the other hand by the therapeutic compliance and the delay of chemotherapy. Indeed, out of 6 to 9 cycles of chemotherapy planned 23% of patients received less than 6 cycles. This is due to the very poor socioeconomic standing of most of our patients. 51% of our patients were from a lower socioeconomic class, whereas the treatment is relatively expensive. Another argument that helped explain this low rate of complete remission was the poor compliance related to treatment (57%). In 43% of cases patients respected the interval between two treatments, which was 21 days. This interval was long in the 57%. Besides, 41% of our patients had an IPI ≥ 3 and therefore several factors of poor prognosis. To improve our rate of complete remission, the institution of immunotherapy with Rituximab combined with CHOP (R-CHOP), which gives complete remission in the range of 80% would be an interesting alternative [4, 19, 20]. But the high cost of this drug makes it out of reach for the majority of our patients. 2nd-generation protocols such as mBACOD (Methotrexate, Bleomycin, Adriamycine, Cyclophosphamide, Vincristine, Dexamethasone) or 3rd-generation protocols such as ACVBP (Adriamycine, Cyclophosphamide, Vincristine, Bleomycine, Prednisone), which provide complete remission rates in the range of 70 to 80%, are difficult to handle in our context of exercise because of the chemo-induced aplasia they cause besides being expensive. CHOP 14 which consists in the administration of CHOP every 14 days is difficult to institute in our practice since it requires growth factors (G-CSF) that increase the cost of treatment [5].

In terms of outcome, we obtained 20 cases of progression free survival, that is 32%, 8 patients were lost to follow up, that is, 13%, and 32 patients died, that is, 51% after 3 years of median follow-up. Our rate of progression-free survival at 3 years, which was 32%, is below the rate in the west, on CHOP. According to the Groupe d'Etudes des Lymphomes de l'Adulte, progression-free survival at 10 years and the global survival at 10 years were, respectively, 20% and 27.6% on CHOP [21]. Our low rate, of progression-free survival at 3 years is related to our complete remission rate which is only 27%. 13% of patients were lost to follow up. These patients, most of the time, financially exhausted deliberately stopped their follow-up.

In terms of analysis, parameters that could influence the complete remission rate were studied. The stage of Ann Arbor, biological b symptoms, the International Prognosis Index (IPI), and the socioeconomic standing are the four parameters that influence the complete remission rate in univariate analysis. In localized stages I and II of Ann Arbor the complete remission rate was 89% whereas this rate dropped to 24% in advanced stages III and IV (*P* < 0.0001). In the group with no of biological b symptoms, the complete remission rate was 65% and 27% in group with yes (*P* < 0.01). The complete remission rate was 83% in the low-risk group versus 22% in the high-risk group (*P* < 0.001). The socioeconomic standing also influences the complete remission rate. This rate was 22% in the low socioeconomic class, 81% in the medium socioeconomic class, and 78% in the high socioeconomic class (*P* = 0.001). The socioeconomic standing, which then constitutes a prognostic factor, is generally one of the particularities of the management of hematologic malignancies in our country. In multivariate analysis, only the IPI (*P* = 0.001) and the stage of Ann Arbor (*P* = 0.01) influence the complete remission rate.

## 5. Conclusion

Diffuse large B-cell lymphoma in black Africans presents some epidemiological, clinical, therapeutic, and prognostic particularities: the age of onset is lower, the disease is more disseminated (stages III and IV of Ann arbor), the complete remission rate is low, the socioeconomic standing constitutes a significant prognostic factor, and some patients are lost to follow up.

## Figures and Tables

**Table 1:** CHOP protocol.

CHOP	Every day	Day
Drugs	Dose and administration route	

Cyclophosphamide	750 mg/m^2^ IV	Day 1
Adriamycin	50 mg/m^2^ IV	Day 1
Vincristine	1.4 mg/m^2^ IV	Day 1
Prednisone	100 mg/m^2^ per oral route	Days 1 to 5

**Table 2:** General characteristics of patients.

Parameters	Number (%) *n* = 63
Age (years), median age: 42 years	
<60	52 (83)
≥60	11 (17)
Sex	
Male	42 (67)
Female	21 (33)
Socioeconomic class	
Low	32 (51)
Medium	22 (35)
High	9 (14)
Site of lymphoma	
Nodal	37 (59)
Extra-nodal	6 (9)
Mixed	20 (32)
Extranodal sites	
Gastrointestinal	7 (11)
Hepatic	6 (10)
Otorhinolaryngology	4 (6)
Pleuropulmonary	4 (6)
Genital	2 (3)
Bone	1 (2)
Stage of Ann Arbor	
I, II	18 (29)
III, IV	45 (71)
IPI	
Low risk (0, 1)	13 (21)
Intermediate low risk (2)	9 (14)
Intermediate high risk (3)	24 (38)
High risk (4, 5)	17 (27)
Clinical B symptoms	
No	16 (25)
Yes	47 (75)
Biological b symptoms	
No	26 (41)
Yes	37 (59)

**Table 3:** Biological characteristics of patients.

Parameters	Number (%) *n* = 63
Hepatic serology	
B positive	3 (5)
C positive	2 (3)
Negative	5 (8)
Not specified	53 (84)
HIV serology	
Positive	11 (17)
Negative	12 (19)
Not specified	40 (64)
LDH rate	
Normal	7 (11)
High	56 (89)
*β*2 microglobulin rate	
Normal	6 (10)
High	57 (90)

**Table 4:** Therapeutic and evolutional characteristics of patients.

Parameters	Number (%) *n* = 63
Type of therapy	
CHOP	63 (100)
RCHOP	0 (0)
Therapeutic response	
Complete remission	27 (43)
Partial remission	25 (40)
Failure	11 (17)
Dose adherence	
Normal	61 (97)
Reduction	2 (3)
Delay of chemotherapy (days)	
<15	44 (70)
15–30	14 (22)
>30	5 (8)
Treatment compliance	
Good	27 (43)
Poor	36 (57)
Toxicity	
Hematological	23 (37)
Metabolic	20 (32)
Digestive	7 (11)
Cardiac	3 (5)
Neuropathies	3 (5)
Alopecia	2 (3)
Number of cycles	
4–7	46 (73)
>7	17 (27)
Outcome at median follow-up	
Progression-free survival	20 (32)
Overall survival	31 (49)
Lost to follow-up	8 (13)
Dead	32 (51)
Causes of death	
Progression	17 (27)
Hematological toxicity	7 (11)
Infection	4 (6)
Metabolic toxicity	2 (3)
Undetermined	2 (3)

**Table 5:** Relationship between the therapeutic response and the general characteristics of patients.

Parameters	Univariate analysis Complete response	*P*
Age (years)		
<60	22/52 (42)	0.552
≥60	5/11 (45)	
Sex		
Male	18/42 (43)	0.787
Female	9/21 (43)	
Socioeconomic standing		
Low	7/32 (22)	**0.001**
Medium	13/22 (59)	
High	7/9 (77)	
IPI		
Low risk (0, 1)	5/6 (83)	**<0.0001**
Intermediate risk (2)	13/16 (81)	
High risk (≥3)	9/41 (22)	
Site		
Nodal	18/37 (49)	0.395
Extranodal	9/26 (35)	
Consultation period (month)		
<3	10/15 (67)	0.06
3–6	10/26 (38)	
>6	7/22 (32)	
Stage of Ann Arbor		
I, II	16/18 (89)	**<0.0001**
III, IV	11/45 (24)	
Clinical B symptoms		
No	8/16 (50)	0.706
Yes	19/47 (40)	
Biological b symptoms		
No	17/26 (65)	** 0.01**
Yes	5/37 (13)	
